# LC–MS-based multivariate statistical analysis for the screening of potential thrombin/factor Xa inhibitors from Radix Salvia Miltiorrhiza

**DOI:** 10.1186/s13020-020-00320-2

**Published:** 2020-04-26

**Authors:** Yi-Yao Yang, Zhao-Yu Wu, Hao Zhang, Shi-Jun Yin, Fang-Bo Xia, Qian Zhang, Jian-Bo Wan, Jian-Li Gao, Feng-Qing Yang

**Affiliations:** 1 School of Chemistry and Chemical Engineering, Chongqing University, Chongqing, 401331 People’s Republic of China; 2 Academy of Chinese Medical Sciences, Zhejiang Chinese Medical University, Hangzhou, Zhejiang 310053 People’s Republic of China; 3 State Key Laboratory of Quality Research in Chinese Medicine, Institute of Chinese Medical Sciences, University of Macau, Macao, People’s Republic of China

**Keywords:** Radix Salvia Miltiorrhiza, Thrombin, Factor Xa, Inhibitor screening, Multivariate statistical analysis, Molecular docking

## Abstract

**Background:**

The dry root and rhizome of *Salvia miltiorrhiza* Bunge, or Danshen, is a well-known traditional Chinese medicine with anticoagulant activity. Taking into account that thrombin (THR) and factor Xa (FXa) play crucial roles in the coagulation cascade, it is reasonable and meaningful to screening THR and/or FXa inhibitors from Danshen.

**Methods:**

Four extracts [butanol (BA), ethyl acetate (EA) and remained extract (RE) from 75% ethanol extract, and water extract (WE)] of Danshen were prepared, and their THR/FXa inhibitory activities were assessed in vitro. Then, the active EA extract was further separated by silica-gel column chromatography (SC), and its fractions (SC1–SC5) were analyzed by LC–MS. The principal component analysis (PCA) and orthogonal partial least squares discriminate analysis (OPLS-DA) were employed for predicting the specific marker compounds. The chemical structures of targeted compounds were identified by LC–MS/MS and their interactions with THR/FXa were analyzed by the molecular docking analysis.

**Results:**

Danshen EA extract showed strong activity against THR and FXa, and its fractions (SC1–SC5) exhibited obvious difference in inhibitory activity against these two enzymes. Furthermore, four marker compounds with potential THR/FXa inhibitory activity were screened by PCA and OPLS-DA, and were identified as cryptotanshinone, tanshinone I, dihydrotanshinone I and tanshinone IIA. The molecular docking study showed that all these four tanshinones can interact with some key amino acid residues of the THR/FXa active cavities, such as HIS57 and SER195, which were considered to be promising candidates targeting THR and/or FXa with low binding energy (< − 7 kcal mol^−1^).

**Conclusions:**

LC–MS combined with multivariate statistical analysis can effectively screen potential THR/FXa inhibitory components in Danshen.

## Background

Radix Salvia Miltiorrhiza, the dry root or rhizome of *Saliva miltiorrhiza* Bunge, namely Danshen in Mandarin, has been used to activate blood circulation to remove blood stasis in traditional Chinese medicine for more than thousands of years. It is widely cultivated in China, such as in Shandong, Sichuan, Henan and Shaanxi provinces [1]. The major bioactive constituents of Danshen can be classified into the hydrophilic salvianolic acids, and lipophilic diterpenoid tanshinones, both of which could contribute to the pharmacological and therapeutic effects of Danshen [2]. The modern pharmacological research showed that Danshen possesses multifarious pharmacological effects such as anticancer [3, 4], anti-inflammatory [5], neuroprotection [6], anti-hypertension [7] and alleviation of diabetic retinopathy [8], etc. And it is one of the most widely applied Chinese medicines in the treatment of cardiovascular and cerebrovascular diseases [9, 10]. However, there are few studies reported about the thrombin (THR) or factor Xa (FXa) inhibitory activity of its extracts or ingredients.

The blood coagulation cascade is a complex and tightly regulated process mediated by plasma protein and cofactors. Employing different coagulation factors as drug targets, coagulation cascade could be destroyed to achieve anticoagulation. Therefore, the coagulation factors’ inhibitors are considered to be the important means to treat thrombotic diseases [11, 12]. THR is a serine protease and closely correlated to thrombosis. As the final effector of coagulation cascade, THR could catalyze the conversion of fibrinogen into insoluble strands of fibrin. It also acts as a potent agonist, which stimulates and recruits platelets to the lesioned site. FXa, which serves as a catalyst in the production of THR by activating prothrombin, is serine proteases at the upstream position from THR and a common mediator of the extrinsic and intrinsic coagulation. Owing to their key roles and unique positions, THR and FXa become the important and ideal targets for the research of anticoagulant drugs. Several clinical available direct THR inhibitors (like argatroban) and FXa inhibitors (like rivaroxaban) still demonstrate flaws such as hemorrhage risk, narrow clinical applications, and so on [13, 14]. On the other hand, the presence of various natural bioactive THR or FXa inhibitors have been reported, including polypeptides [15–18], polyphenols [19, 20], saponins [21] and other compounds [22–24], because of natural products have the properties of wide source, structural and bioactive diversities. Therefore, it is reasonable to screening THR or FXa inhibitors with less side effects from natural products such as Danshen.

The multivariate statistical analysis method can process huge amount of liquid chromatography paired with mass spectrometry (LC–MS) data and rapidly identify the differences among sample groups [25]. When it was combined with bioactivity assay, the method can simplify the isolation process of phytochemistry and effectively determine the components that contribute to the pharmacological activity of the natural product [26]. This method has been proved feasible and effective in recent years, such as being employed to identify antidiabetic compounds from Ge-Gen-Qin-Lian decoction [27], screen antiplatelet chemical compositions of edible *Citrus limon* [28] and analyze antioxidant marker compounds from blueberries [29].

Therefore, an LC–MS-based multivariate statistical analysis method was reported in this study for the screening of potential THR/FXa inhibitors from Danshen. Firstly, the THR and FXa inhibitory activities of different Danshen fractions were compared. Then, to visualize the chemical difference and predict the components (marker compounds) responsible for inhibiting THR/FXa, the principal component analysis (PCA) and orthogonal partial least squares discriminant analysis (OPLS-DA) were conducted on the MS data of Danshen fractions correlating with enzyme inhibitory activity. Finally, molecular docking was utilized to further confirm the binding sites of marker compounds with THR/FXa and to predict other possible enzyme inhibitors, which have similar structure characteristics to the screened out compounds.

## Materials and methods

### Plant materials and reagents

Crude drug of Danshen used in this study was purchased from Chongqing Xinhu Pharmacy Co., Ltd. (Chongqing, China), and the voucher specimen (No. DS2019033001) was deposited at the Pharmaceutical Engineering Laboratory in the School of Chemistry and Chemical Engineering, Chongqing University, Chongqing, China. The sample was authenticated using morphological characters as described in the Chinese Pharmacopoeia (2015 edition) (Committee, NP 2015).

Dimethyl sulphoxide (DMSO), Tris (hydroxymethyl) aminomethane (Tris) were obtained from Sangon Biotech Co., Ltd (Shanghai, China). Argatroban was purchased from Harvey-bio Co., Ltd (Beijing, China). Dopamine hydrochloride and rivaroxaban were purchased from Aladdin Chemistry Co., Ltd (Shanghai, China). FXa, S-2238 and S-2765 all were products of Adhoc International Technologies Co., Ltd (Beijing, China), and THR was bought from Sigma-Aldrich (St Louis, USA). HPLC-grade methanol and formic acid were obtained from Shanghai Tedia Scientific Co., Ltd (Shanghai, China). Water used for all the experiments was purified by a water purification system (ATSelem 1820A, Antesheng Environmental Protection Equipment Co., Ltd, Chongqing, China). Unless otherwise specified, all other chemicals and solvents, such as ethanol, butanol (BA), ethyl acetate (EA), petroleum ether (PE), sodium hydroxide (NaOH), hydrochloric acid (HCl), sulphuric acid (H_2_SO_4_) and vanillin were of analytical grade and purchased from Chron Chemicals Co., Ltd (Chengdu, China).

The running buffer containing 10 mM Tris was adjusted to pH 8.0 with 1 M HCl. All samples were prepared by dissolving the respective substance in DMSO and diluted with Tris buffer (10 mM, pH 8.0) to the required concentrations for THR/FXa inhibitory assay, which were stored at 4 °C and shielded from light before use. THR was dissolved in Tris buffer (10 mM, pH 8.0) with the enzyme activity of 500 U mL^−1^, and stored at − 20 °C. FXa was also dispensed in Tris buffer (10 mM, pH 8.0) with the enzyme activity of 0.5 IU mL^−1^, and stored at 4 °C. The substrates include S-2238 and S-2765 were prepared by dissolving each compound in Tris buffer and the concentration was 2.5 mg mL^−1^, respectively.

### Preparation of sample extracts

After comminution, 100 g of Danshen powder was accurately transferred into a 2 L glass-stoppered conical flask, and then was extracted with 800 mL 75% ethanol (1:8, w/v) for 1 h in a water bath at 80 °C; then the extract was filtered, and the residue was collected. The above process was repeated for two times. Three extract solutions were combined and concentrated in a rotavapor (ZFQ 85 A, Shanghai Medical Instrument Special Factory, Shanghai, China) at 45 °C. After removing ethanol completely, the concentrate was degreased with petroleum ether (2:1, v/v), and further subjected to liquid–liquid partitioning to afford EA—(2:1, v/v), BA—(1:1, v/v) and remained extract (RE). Then, removed the solvent by reduced pressure distillation and vacuum dry method (DZF-6050, Shanghai Jing Hong Laboratory Instrument Co., Ltd., Shanghai, China), three portions were obtained. In addition, 600 mL water (1:6, w/v) was added to the residues for extraction twice on water bath at 80 °C. These two supernatant was combined and evaporated, and was further vacuum-dried. Finally, the water extract (WE) was obtained. With the purpose of further HPLC and LC–MS analyses, these extracts were made into methanol solution (0.5 mg mL^−1^), and were filtered through a 0.22 μm nylon membrane (Shanghai Titan Scientific Co., Ltd., Shanghai, China).

### In vitro THR/FXa inhibitory activity assays

Thrombin inhibitory activity assays were carried out on an Agilent 7100 3^D^ capillary electrophoresis (CE) system (Agilent Technologies, Palo Alto, CA, USA), which equipped with a diode array detector and Agilent ChemStation software. All of the experimental procedures were implemented according to literatures with minor modifications [30]. The first step was the preparation of immobilized enzyme microreactor (IMER). A new bare fused-silica capillary (75 μm, purchased from Yongnian Ruifeng Chromatographic Device Co., Ltd., Hebei, China) was flushed sequentially with 1 M NaOH for 15 min and deionized water for 10 min. An automated program was set to prepare the IMER: The dopamine solution (2 mg mL^−1^) was introduced into the capillary with a voltage of + 10 kV for 10 s, stayed for 30 min, and then using running buffer (10 mM Tris–HCl buffer solution, pH 8.0) with a pressure of − 100 mbar for 90 s to wash out the free dopamine. Then, the THR solution (125 U mL^−1^) was injected into the capillary with a voltage of + 10 kV for 10 s, kept for 30 min; and then was flushed by running buffer with a pressure of − 100 mbar for 90 s to flush out free THR. The prepared IMER can be used immediately for THR inhibitory activity assay. The second step was inhibition study of THR on of the prepared IMER: The ambient temperature of the capillary cartridge was maintained at 25 °C and the detection wavelength was set at 405 nm. To carry out the enzyme inhibitory activity assays, the substrate solution (2.5 mg mL^−1^) with/without inhibitors was injected into the inlet of IMER at a voltage of + 10 kV for 10 s and incubated for 60 s to trigger amidolytic reaction. The voltage of + 25 kV was applied to separate all the reaction mixtures with the aim of p-Nitroaniline detection. The FXa inhibitory activity assays (0.5 IU mL^−1^) were measured in the same manner as THR, but using S-2765 as substrate. The inhibition percentage was calculated by the formula:

1 $${\text{Inhibition percentage}}\left( {\text{\% }} \right) = \left( {1 - \frac{{A_{sample} }}{{A_{blank} }}} \right) \times 100$$

where $A_{blank}$ and ${\text{A}}_{sample}$ are the peak area of product gained by enzymatic reaction of the blank and sample group, respectively. All assays were performed in triplicate and the inhibition ratios were the mean of three observations.

### HPLC-DAD analysis

An Agilent 1260 Series liquid chromatography system (Agilent Technologies, Palo Alto, California, USA) was equipped with a vacuum degasser, a binary pump, an autosampler, and a diode array detector (DAD) and system control and data analysis were processed with the Agilent ChemStation software. The separation was performed on an Agilent ZORBAX SB-C_18_ column (150 × 4.6 mm i.d., 5 µm) and a pre-column (ZORBAX SB-C_18_ guard column, 12.5 × 4.6 mm i.d., 5 μm). The binary mobile phase was composed with solvent A (0.1% aqueous formic acid) and solvent B (methanol) with a gradient elution program: 0–10 min, 20–55% B; 10–20 min, 55–65% B; 20–30 min, 65–70% B; 30–50 min, 70–80% B. The solvent flow rate was 0.8 mL min^−1^, the DAD detection wavelength was set at 273 nm, the column was maintained at 30 °C and the injection volume was 10 µL.

### LC-SQD–MS analysis

The LC–MS analysis was conducted on an electrospray ionization mass spectrometer (ESI-MS) consisting a single quadrupole detector (SQD) as the mass detector (Waters, Milford, MA, USA), which was equipped with a UPLC system. The LC conditions were the same as described above. The ESI-MS conditions were as follows. ESI was used in both the positive and negative mode. Nitrogen gas was used for desolvation at a flow rate of 550 L h^−1^ at 350 °C. The capillary voltage was 3000 V, the temperature of the ionization source was 100 °C, and the cone voltage was 30 V. The MS data were recorded in the full scan mode (*m*/*z* 100–800).

### LC–MS/MS identification

The LC–MS/MS identification was conducted on Shimadzu LC/MS–MS 8060 electrospray ionization-mass spectrometer, consisting of a triple quadruple detector as the mass detector (Shimadzu, Kyoto, Japan) and coupled with HPLC via a PEEK tube (0.13 mm i.d.). LC conditions were the same as that described in “HPLC-DAD analysis” section. The mass spectrometric parameters were set as below: the mass spectra were recorded in positive mode; drying gas was set at a flow rate of 10 L min^−1^ at 400 °C; curved desolvation line voltage was set at constant level; nebulizing gas was nitrogen and the flow rate was set at 3 L min^−1^; block heater temperature, 250 °C; MS^1^ data was recorded in the full-scan mode and the mass scan range was from *m*/*z* 100 to 1000, and MS^2^ data was recorded in product ion scan mode. Data acquisition and processing was performed with the LC–MS solution version 1.1 software package (Shimadzu).

### Data processing and multivariate analysis

The raw LC-SQD–MS data were extracted and processed using the Progenesis QI software (Waters Corporation, Milford, MA, USA). Peak detection, alignment, peak integration and retention time correction were carried out with a *t*_R_ window of 0.1 min and a mass window of 0.05. The apex track peak detection parameters were utilized to automatically detect the peak width and baseline noise. The raw data had not undergone smoothing processing. The used parameters included a retention time range of 0–50 min, a mass range of 100–800 Da, a noise elimination level of 6%. Through applying above parameters, the ions from different samples were considered to be the same when they possessed the same *t*_R_ (tolerance of 0.1 min) and *m*/*z* (tolerance of 0.05 Da) values. The intensity of each ion was normalized with respect to the total ion count to generate a resultant three-dimensional data matrix that consisted of the retention time, *m*/*z* value, and the normalized ion intensities. With the aim of reducing the effect of noise in the chromatograms, all the variables were *pareto*-scaled prior to PCA and OPLS-DA by SIMCA-P^+^ 13.0 Software (Umetrics, Umeå, Sweden). As an unsupervised analytical method, PCA rapidly provides a first overview of understanding the integrity view of hidden information from the sample data. OPLS-DA is a supervised analytical method and usually used to modelling two or more classes of data to provide a good class separation, simplified interpretation, and reveal potential biomarkers contributing to intergroup difference [31].

### In silico molecular docking of THR/FXa and identified active compounds

In silico molecular docking simulations were carried out by Auto Dock 4.2 program (The Scripps Research Institute, La Jolla, CA, USA) to validate the binding potency of the compounds to THR [32]. The docking operation was conducted according to the following steps: First, prepare the file of receptor protein. Download the X-ray co-crystal structure file of THR-argatroban complex from Protein Data Bank database (PDB code = 1DWC, resolution of 3 Å; organism, *Homo sapiens*) [33], following with the deletion of unnecessary water molecules and the ligand argatroban, and addition of polar hydrogen atoms. Second, prepare the file of compounds. Chemoffice 3D was performed to drawn the 3D chemical structure of marker compounds and output in PDB format with minimized energy. Third, the grid size was set to (x, y, z) = (60, 60, 60), and the catalytic site of the grid box was set to (x, y, z) = (35.887, 19.178, 18.856). In each simulation process, progress with default parameters run from Autogrid and Autodock. With the aim of finding the most favorable ligand binding orientations, Lamarckian genetic algorithm (LGA) was employed and the running times of GA was set to 50 for giving docked conformations. The interaction figures were generated, and the docking results of the small molecules binding to the active pocket of THR were recorded with binding energies and bonded residues. Furthermore, the 2D interaction diagrams were obtained by Discovery Studio 4.5 (Dassault Systèmes BIOVIA, San Diego, CA, USA). To validate the binding potency of the compounds to FXa, the same steps were repeated with the different parameters: using the crystal structure of FXa-rivaroxaban complex (PDB code = 2W26, resolution = 2.08 Å) [34]; the catalytic site of grid box was set to (x, y, z) = (7.951, 5.850, 21.876). The other details were kept constant.

## Results

### Bioactivity-guided fractionation

**Fig. 1:**
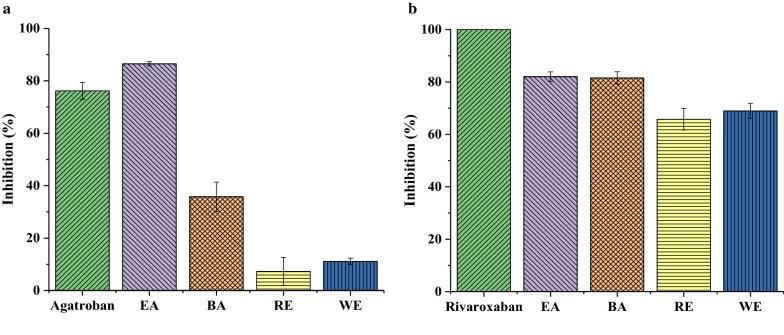
Inhibitory activity of different fractions of Danshen crude extract on the THR (**a**) and FXa (**b**)

The inhibitory activities against THR/FXa of the EA, BA, RE and WE extracts of Danshen (1.5 mg mL^−1^) and each positive control, argatroban and rivaroxaban (0.5 mg mL^−1^) were assessed, respectively. The results were shown in Fig. 1. The EA extract showed the strongest inhibitory activity toward these two enzymes and was chosen for further fractionation.

**Fig. 2:**
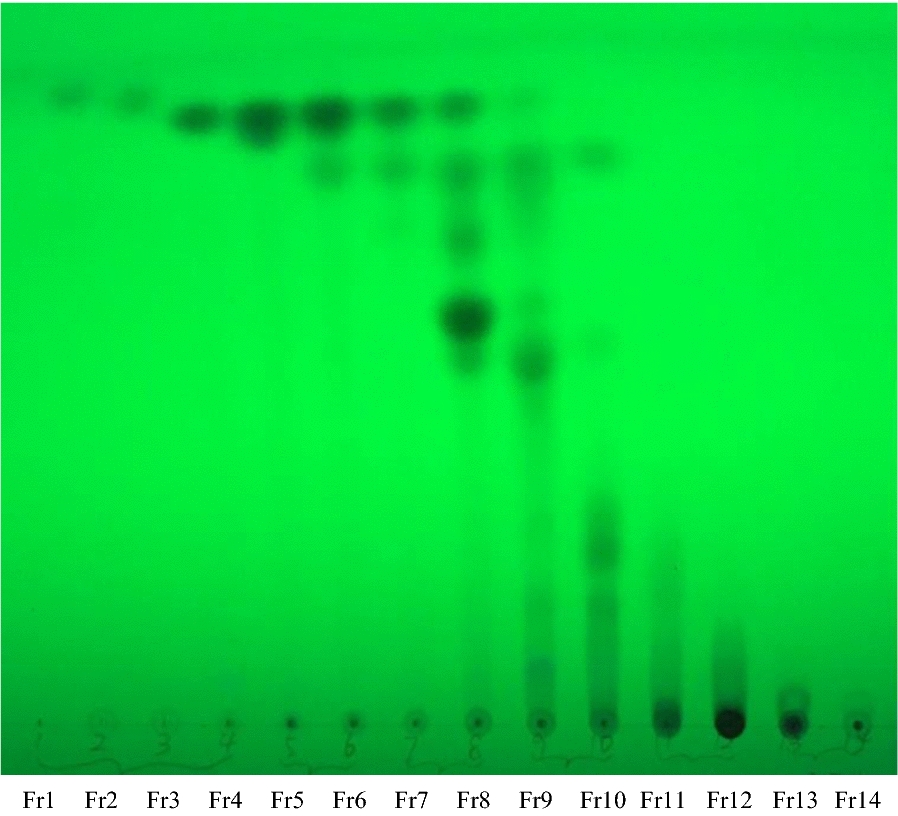
TLC analysis of Danshen 14 fractions under ultraviolet light (UV 254 nm)

**Fig. 3:**
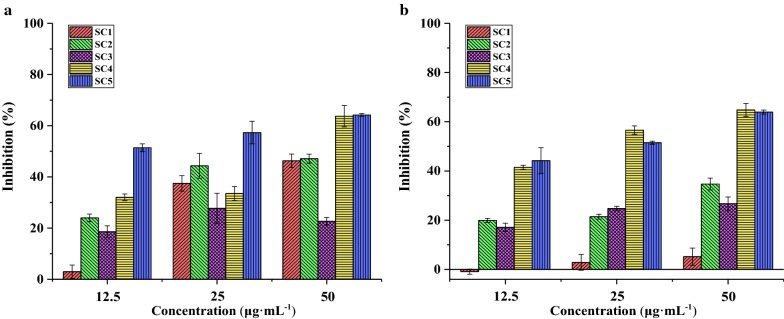
Inhibitory activity of different silica gel fractions (SC) of Danshen EA extract on the THR (**a**) and FXa (**b**)

A total of 4.15 g EA extract (prepared from 400 g Danshen powder) was applied to normal silica gel column chromatography (SC), and was eluted with gradient of PE-EA (10:1 to 1:2) and 100% EA. The obtained 14 SC fractions (Fr1–Fr14) were recombined based on thin-layer chromatography (TLC) analysis, which was shown in Fig. 2. Similar fractions, as Fr1–Fr4, Fr5–Fr7, Fr8–Fr9, Fr10–Fr12 and Fr13–Fr14, were grouped, and contiguous mixture had common components. Then removed the solvent and five fractions were yielded (SC1–SC5). Likewise, a THR activity evaluation test was employed to examining activity differences of these five fractions (Fig. 3a). Fraction SC4 (1.254 g) and SC5 (0.437 g) exhibited similar activity that both had the strongest inhibitory effect in the THR inhibitory activity assay, and both fractions SC1 and SC2 were shown moderate activity, while SC3 exhibited a weak effect. Moreover, as shown in Fig. 3b, the result of FXa activity evaluation test among five fractions indicated that these five fractions could be classified into the most active (SC4 and SC5), moderate active (SC3 and SC2) and low active (SC1) groups.

### Multivariate statistical analysis of active compounds from different fractions

**Fig. 4:**
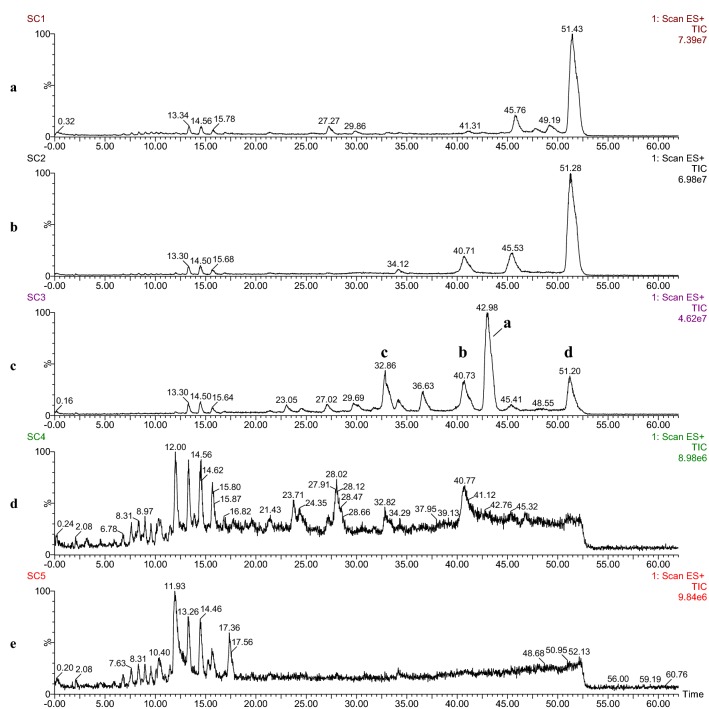
Total ion chromatograms of five Danshen fractions SC1 (**a**), SC2 (**b**), SC3 (**c**), SC4 (**d**) and SC5 (**e**)

LC-SQD–MS analysis provided raw data on the constituent profiles of sequential Danshen fractions separated by silica gel column chromatography. Considering that the total ion chromatography under the positive ion detection mode owned less fragmentation ions and much clearer chromatographic peaks with less background interference, the MS data of each biological samples under positive ion mode was collected prior to multivariate statistical analysis. The parameters setting in the acquisition of raw data in the positive mode: a scan time of 0.05 s with an inter-scan time of 0.01 s and a polarity switch time of 0.3 s. The representative total ion chromatographs of five SC fractions (SC1–SC5) were illustrated in Fig. 4. The processed LC–MS data matrix was generated by Progenesis QI and then was subjected to multivariate statistical analysis, including PCA and OPLS-DA.

**Fig. 5:**
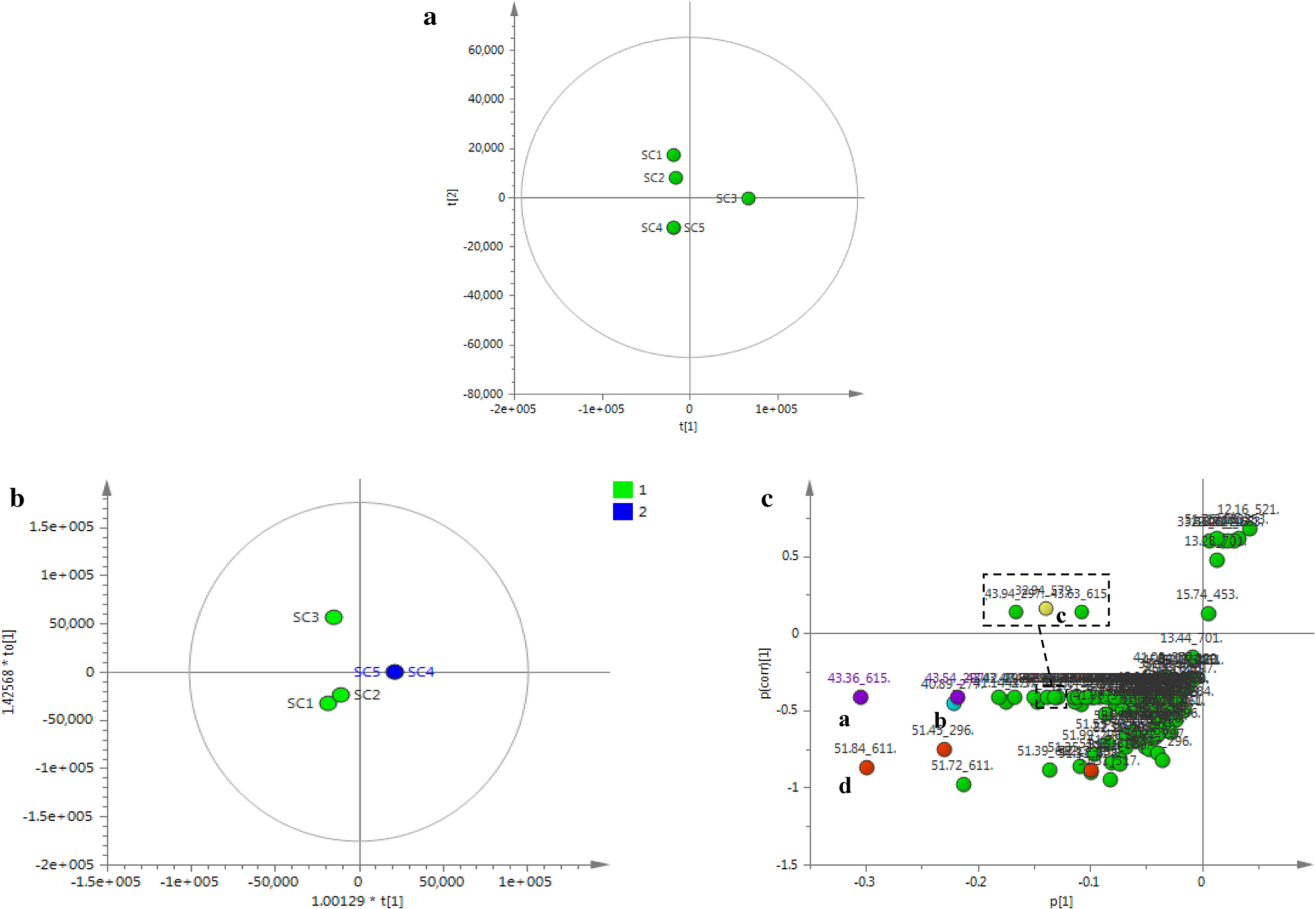
Chemometric analysis of five Danshen fractions. Score plot from PCA (**a**), Score plot (**b**) and S-plot (**c**) from OPLS-DA with a Hotelling’s 95% confidence ellipse. Marker a: purple, Marker b: blue, Marker c: yellow, Marker d: orange

With the aim of assessing differences in the chemical constituents of each fraction, non-targeted PCA was employed to visualization the clustering and trends by loading score plot; the closer the points in the PCA score plot, the more similar the sample data are. As shown in Fig. 5a, in the PCA scores plot of Danshen EA fractions, SC4 and SC5 were separated into a cluster distinct from other fractions, and the other two groups were observed corresponding with the results of THR/FXa inhibitory activity assays in a certain degree. The values of the established PCA model fit parameters *R*^2^*X* (cum) and *Q*^2^ (cum) were 0.960 and 0.896, respectively, which indicated that the model is robust [35].

In order to further investigate the potential marker compounds, sample MS data were set divided classes based on the cluster from PCA score plot and the difference of enzyme inhibitory activity (active, less active), and then subjected to a supervised discriminant analysis, OPLS-DA. The OPLS-DA, which is an applicable method for revealing differential markers, could distinguish chemical patterns. The fitted OPLS-DA model exhibited good fitness (*Q*^2^ (cum) 0.926) and predictability (*R*^2^*X* (cum) 0.96, *R*^2^*Y* (cum) 0.984). All the observations fell within the Hotelling T2 (0.95) ellipse. The OPLS-DA score plot is presented in Fig. 5b, and the five fractions are clearly distinguished and could be classified as active (SC4, SC5) and less active (SC1–SC3). The S-plot generated from OPLS-DA displayed the ions conducive to distinguishing groups of the Danshen fractions. The correlations within the same sample group (*p* [corr]) were expressed by the vertical Y-axis and the variable contributions (*p* [1]) were represented by the horizontal X-axis. Thus, the ion points closer to the lower left and upper right corners made a larger contribution to the observed separation of the samples in OPLS-DA [36]. These markers may be associated with the enzyme inhibitory activity. The S-plots of two OPLS-DA models were shown in Fig. 5c. The points at the two ends of “S” with high variable importance in the projection (VIP) scores (VIP > 1), were selected and named as a–d. The detailed information was listed in Table 1.

**Table 1:** Detail information of four marker compounds obtained from OPLS-DA S-plot

Marker compounds	*t*_R_ (min)-*m*/*z*	Ions	VIP	Formula (neutral form)	MS/MS fragments	Identification
a	43.54–297^a^	[M+H]^+^	2.63	C_19_H_20_O_3_	279, 264, 251, 223, 208	Cryptotanshinone
	43.36–615^a^	[2M+Na]^+^	3.67			
b	40.89–277^a^	[M+H]^+^	2.74	C_18_H_12_O_3_	259, 249, 231, 221, 206, 193, 178	Tanshinone I
	40.87–575^b^	[2M+Na]^+^				
c	32.82–279^b^	[M+H]^+^		C_18_H_14_O_3_	218, 205, 190	Dihydrotanshinone I
	32.94–579^a^	[2M+Na]^+^	1.57			
d	52.17–295^a^	[M+H]^+^	1.28	C_19_H_18_O_3_	277, 262, 249, 234, 221, 206, 191	Tanshinone IIA
	51.45–296^a^	Isotope peak of [M+H]^+^	2.62			
	51.74–317^b^	[M+Na]^+^				
	51.84–611^a^	[2M+Na]^+^	3.60			

^a^ Represents the ion screened from S-plot of OPLS-DA model

^b^ Represents the ion that found in MS data to assist identification

### Mass fragmentation analysis of marker compounds

**Fig. 6:**
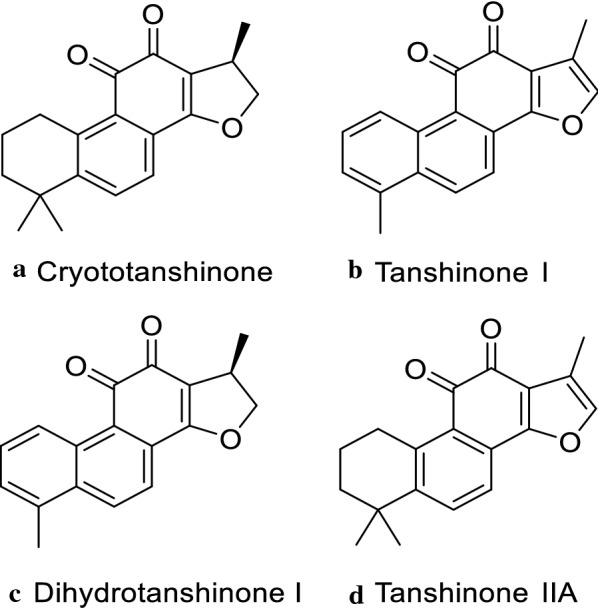
Chemical structures of marker compounds screened from Danshen extract

In order to identify the chemical markers a–d, LC–MS/MS analysis was utilized to perform the MS^2^ data verification with the aid of previously reported literature [37–40]. Phenolic acids and diterpenes are the main components in Danshen crude drug [41], and most of the chemical compositions in EA fraction from Danshen were low polarity components (diterpenoid tanshinones), thus, positive ion mode was tried in LC–MS/MS analysis. By comparing the retention time, fragmentation behaviors and MS data (Table 1) of the peaks in the tested samples, four compounds [(a) cryptotanshinone, (b) tanshinone I, (c) dihydrotanshinone I and (d) tanshinone IIA) were tentatively identified, and the chemical structures of these screened compounds were shown in Fig. 6.

Marker a showed a peak of [M+H]^+^ ion at *m*/*z* 297, [M+Na]^+^ ion at *m*/*z* 319 and [2M+Na]^+^ at *m*/*z* 615. The fragment at *m*/*z* 279 indicates the loss of a neutral substituent [M+H–H_2_O]^+^ and sequential cleavage to produce the fragments of [M+H–H_2_O–CH_3_]^+^ at *m*/*z* 264, [M+H–H_2_O–CO]^+^ at *m*/*z* 251, [M+H–H_2_O–2CO]^+^ at *m*/*z* 223 and [M+H–H_2_O–2CO–CH_3_]^+^ at *m*/*z* 208 by losing the CH_3_ or CO. Therefore, marker a was identified as cryptotanshinone [37, 38, 40]. Marker b displayed [M+H]^+^ ion at *m*/*z* 277, [M+Na]^+^ ion at *m*/*z* 299 and [2M+Na]^+^ ion at *m*/*z* 575. The [M+H]^+^ ion at *m*/*z* 277 produced [M+H–H_2_O]^+^ (*m*/*z* 259) and [M+H–CO]^+^ (*m*/*z* 249). In addition, ions at *m*/*z* 221, *m*/*z* 193 and *m*/*z* 178 were found, and were formed by sequential losses of CO, CO and CH_3_ from [M+H–CO]^+^. Therefore, marker b was identified as tanshinone I [37, 38]. Marker c gave [M+H]^+^ ion at *m*/*z* 279, [M+Na]^+^ ion at *m*/*z* 301 and [2M+Na]^+^ ion at *m*/*z* 579. The product ions were obtained at *m/z* 218 for [M+H–H_2_O–CO–CH_3_]^+^, *m/z* 205 for [M+H–H_2_O–2CO]^+^ and *m/z* 190 for [M+H–2H_2_O–2CO]^+^. Therefore, marker c was identified as dihydrotanshinone I [39]. Marker d yielded [M+H]^+^ ion at *m*/*z* 295, [M+Na]^+^ ion at *m*/*z* 317 and [2M+Na]^+^ ion at *m*/*z* 611. Ions at *m*/*z* 249, *m*/*z* 221 and *m*/*z* 206 were also detected, which were formed by sequential losses of CO, CO and CH_3_ from [M+H–H_2_O]^+^ ion (*m*/*z* 277). Therefore, marker d was identified as tanshinone IIA [39, 40].

### Molecular docking analysis of THR/FXa and identified active compounds

**Fig. 7:**
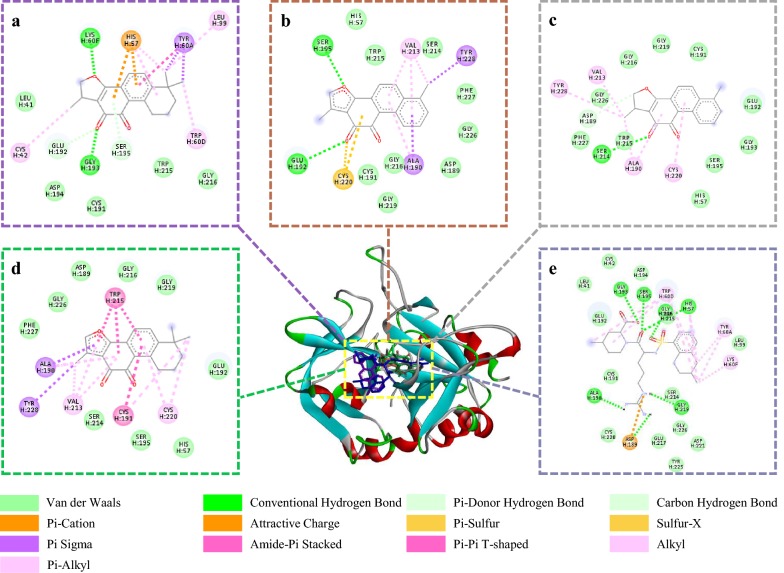
Comparison of 3D structures of four screened inhibitors docked with the THR catalytic site. **a** Cryptotanshinone; **b** tanshinone I; **c** dihydrotanshinone I; **d** tanshinone IIA; **e** argatroban

**Fig. 8:**
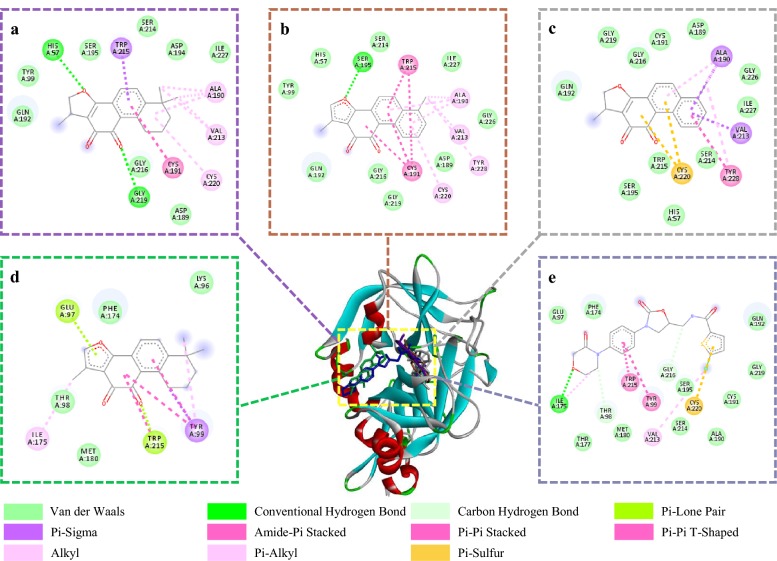
Comparison of 3D structures of four screened inhibitors docked with the FXa catalytic site. **a** Cryptotanshinone; **b** tanshnione I; **c** dihydrotanshinone I; **d** tanshinone IIA; **e** rivaroxaban

Molecular docking studies are widely used to predict the binding mechanism between compounds and the protein targets. In this study, the components screened from Danshen extract and some compounds with similar structure to them were docked with THR or FXa, respectively. The docking energy and binding residues were summarized in Tables 2, 3. The 2D interaction diagrams of screened compounds with residues of THR/FXa can be observed in Figs. 7, 8, respectively. Based on the docking results, all the screened inhibitors could insert into the catalytic active pocket of THR/FXa like original ligand, and combine well with two enzymes through diverse interactions such as hydrogen bond and van der Waals, etc. The active sites of THR and FXa have four binding pockets [42, 43]: S1 pocket (specificity pocket), S2 pocket (proximal pocket), S3 pocket, and S4 pocket (aryl binding pocket). For the docking with THR, the main part of argatroban interacted with S2 pocket and partially blocked S1 pocket with the guanido group. Cryptotanshinone could occupy the S2 pocket and the other six tanshinones were mainly located at S1 pocket. Take tanshinone I for example, it could insert into S1 pocket by interacting with ASP189, GLY216, GLY219, CYS191, GLY226, PHE227, SER214 (van der Waals), CYS220, ALA190, TYR228, VAL213 (electrostatic interaction, EI), bound to S2 pocket by forming interaction with SER195 (Hydrogen bond), HIS57 (van der Waals), and bound to S4 Pocket via TRP215 (van der Waals). For the docking with FXa, rivaroxaban were mainly located at the S4 pocket, and its chlorothiophene carboxamide was interacted with amino acids between S1 and S2 pockets. Tanshinone IIA could occupy the S4 pocket and the other six tanshinones were mainly inserted into S1 pocket. For example, tanshinone I could insert into S1 pocket by interacting with ASP189, GLY216, GLY219, GLY226, ILE227, SER214 (van der Waals), ALA190, CYS191, CYS220, TYR228, VAL213 (EI), bound to S2 pocket by forming interaction with SER195, GLU192 (Hydrogen bond), HIS57 (van der Waals), and bound to S3 and S4 Pocket via TRP215 (EI), TYR99, GLN192 (van der Waals).

**Table 2:** Docking results and residues interactions of seven tanshinones with THR

Compounds	Docking energy (kcal mol^−1^)	Hydrogen bond	Van der Waals	Electrostatic interaction
Cryptotanshinone	− 7.76	LYS60F, GLU192, GLY193, SER195	LEU41, GLY216, TRP215, CYS191, ASP194	TYR60A, HIS57, CYS42, TRP60D, LEU99
Tanshinone I	− 8.21	SER195, GLU192	HIS57, TRP215, SER214, PHE227, GLY226, ASP189, GLY216, CYS191, GLY219	SER195, CYS220, ALA190, TYR228, VAL213
Dihydrotanshinone I	− 7.80	ASP189, SER214	GLY226, GLY216, GLY219, CYS191, GLU192, GLY193, SER195, HIS57, TRP215, PHE227	CYS220, VAL213, TYR228, ALA190
Tanshinone IIA	− 8.07	–	PHE227, GLY226, ASP189, GLY216, GLY219, GLU92, HIS57, SER195, SER214	CYS220, TRP215, ALA190, TYR228, VAL213, CYS191
Tanshinone IIB	− 8.06	SER195, ASP189, GLY219	HIS57, GLY193, GLU192, GLY216, GLU217, CYS220, TRP215, PHE227, GLY226	TYR228, VAL213, ALA190, SER214, CYS191
Methyltanshinonate	− 8.63	GLY219	SER195, HIS57, PHE227, SER214, GLY226, ASP189, GLY216, GLY217, GLU192	ALA190, VAL213, TYR228, TRP215, CYS220, CYS191
Trijuganone B	− 8.42	GLY226, PHE227	SER195, GLU192, GLY193, CYS220, GLU217, GLY219, GLY216, ASP189, SER214, VAL213, TRP215	CYS191, ALA190, TYR228
Argatroban	− 8.90	GLY219, GLY216, HIS57, SER195, GLY193, ALA190	ASP221, CYS220, CYS191, ASP194, GLU217, TRP215, GLY226, SER214, GLU192, LEU99, CYS42, LEU41, TYR225	LYS60F, TRP60D, ASP189, TYR60A

**Table 3:** Docking results and residues interactions of seven tanshinones with FXa

Compounds	Docking energy (kcal mol^−1^)	Hydrogen bond	Van der Waals	Electrostatic interaction
Cryptotanshinone	− 7.52	HIS57, GLY219	GLN192, TYR99, SER195, SER214, ILE227, GLY216, ASP189, ASP194	ALA190, CYS191, CYS220, TRP215, VAL213
Tanshinone I	− 8.26	SER195	GLN192, TYR99, HIS57, SER214, ILE227, GLY226, ASP189, GLY216, GLY219	TRP215, CYS191, CYS220, ALA190, TYR228, VAL213
Dihydrotanshinone I	− 8.12	–	ASP189, GLY226, GLY216, GLY219, CYS191, GLN192, GLY193, SER195, SER214, HIS57, TRP215, ILE227	CYS220, VAL213, TYR228, ALA190
Tanshinone IIA	− 7.73	–	PHE174, THR98, MET180, LYS96	GLU97, ILE175, TRP215, TYR99
Tanshinone IIB	− 7.77	GLN192, GLY216, SER195, ASP194, GLY193, GLY219	HIS57, SER214, GLU217, CYS220, TRP215	TYR99, VAL213, PHE174, CYS191
Methyltanshinonate	− 8.37	SER214, SER195, GLY193, GLN192, ALA190	TYR99, ILE227, CYS220, GLY226, ASP189, ALA221, GLY216, GLY219	HIS57, TRP215, VAL213, TYR228, CYS191
Trijuganone B	− 8.63	–	SER195, GLN192, GLY226, CYS220, ALA221, GLY219, GLY216, ASP189, SER214, TYR99, HIS57, ILE227	CYS191, ALA190, TYR228, TRP215, VAL213
Rivaroxaban	− 10.19	GLY216, THR98, ILE175	CYS191, GLU97, GLN192, MET180, PHE174, SER195, SER214, THR177, ALA190, GLY219	CYS220, TRP215, TYR99, VAL213

## Discussion

Danshen is a well-known traditional Chinese medicine that has been reported to display properties of activating blood circulation to remove blood stasis in the clinic. In this study, the active THR/FXa inhibitors of Danshen were analyzed through multivariate statistical analysis approach (PCA and OPLS-DA), a powerful tool that has emerged to simply and rapidly screen the marker compounds from natural products.

It was observed that Danshen EA extracts had good inhibitory activity against both THR and FXa, and its fractions (SC1–SC5) exhibited certain difference of the activity. Thus, the chemical profiles of five fractions were compared using multivariate statistical analysis. Based on OPLS-DA model, four marker components [(a) cryptotanshinone, (b) tanshinone I, (c) dihydrotanshinone I and (d) tanshinone IIA) with potential THR and FXa inhibitory activity were screened from Danshen via S-plot and VIP value. The results indicated that Danshen has the same effective components toward THR and FXa, which might be the similarity of chemical difference among the activity-based grouping [between high polar components (SC4 and SC5) and low polar components (SC1–SC3)].

In addition, molecular docking was further employed to studying the binding mechanism of the screened tanshinones and some compounds with similar structure to them with THR or FXa. These tanshinones constitute the main liposoluble components in Danshen [38]. According to the structural biology of serine proteases, ASP102, HIS57 and SER195 form catalytic triad. S1 and S4 pocket, together with ASP102-HIS57-SER195 triad, were typically explored for obtaining high-affinity FXa inhibitors [44]. The chemical footprint of S1 pocket of THR and FXa was almost the same, and their S2 and S4 pocket were hydrophobic, which gave the clue of dual inhibitors. All of the screened components could bind with these crucial sites. Moreover, it was usually considered that the region with binding energy under − 5.0 kcal mol^−1^ could be regarded as the potential targets [45]. The binding energy of seven tanshinones were all less than − 7.0 kcal mol^−1^, which suggested that these tanshinones having potential to be THR/FXa inhibitors or dual inhibitors.

## Conclusions

This study successfully screened the THR and/or FXa inhibitors from Danshen by a THR/FXa inhibitory activity assays with a LC-SQD–MS-based multivariate statistical analysis method. Four screened inhibitors, tanshinone IIA, cryptotanshinone, tanshinone I, and dihydrotanshinone I were identified, which are the main active ingredients of Danshen [1]. Meanwhile, docking results showed that screened tanshinones and some compounds with similar structure to them (tanshinone IIB, methyltanshinonate, trijuganone B) had low binding energy. These compounds could bind to catalytically active site of THR and FXa, which are considered to be possible THR and/or FXa inhibitors. These results enriched the cognition of the anticoagulation mechanisms of Danshen extract. This study is the first report of LC-SQD–MS-based multivariate statistical analysis for the screening of bioactive THR and/or FXa target marker compounds from natural products. The present approach is likely time-saving and reagent-conserving compared with activity-guided phytochemical separation method, and could be further applied to the prediction of active components with THR or FXa inhibitory activity in other traditional Chinese medicines. This information will also be helpful in providing a reference for the discovery of novel active THR and/or FXa inhibitors.

## Data Availability

The research data generated from this study is included within the article.

## Abbreviations

BA: Butanol
CE: Capillary electrophoresis
DAD: Diode array detector
DMSO: Dimethyl sulphoxide
EA: Ethyl acetate
EI: Electrostatic interaction
ESI-MS: Electrospray ionization-mass spectrometer
FXa: Factor Xa
IMER: Immobilized enzyme microreactor
LC–MS: Liquid chromatography paired with mass spectrometry
LGA: Lamarckian genetic algorithm
OPLS-DA: Orthogonal partial least squares discriminant analysis
PCA: Principal component analysis
PE: Petroleum ether
RE: Remained extract
SC: Silica gel column chromatography
SQD: Single quadrupole detector
THR: Thrombin
TLC: Thin-layer chromatography
Tris: Tris (hydroxymethyl) aminomethane
VIP: Variable importance in the projection
WE: Water extract
